# Patient discourse on chronic kidney disease monitoring: a qualitative study at a Veterans Affairs Renal Clinic

**DOI:** 10.1186/s12882-018-0981-7

**Published:** 2018-07-19

**Authors:** Ann E. Vandenberg, Katharina V. Echt, Theodore M. Johnson, C. Barrett Bowling

**Affiliations:** 1 Birmingham/Atlanta Geriatric Research, Education, and Clinical Center, Department of Veterans Affairs, Atlanta VA Medical Center, Decatur, GA USA; 2 Department of Medicine, Division of General Medicine and Geriatrics, Emory University School of Medicine, Atlanta, GA USA; 3 Durham Geriatric Research, Education, and Clinical Center, Department of Veterans Affairs, Durham VA Medical Center, Durham, North Carolina USA; 4 Department of Medicine, Duke University School of Medicine, Durham, North Carolina USA

**Keywords:** Health communication, Chronic kidney disease, Chronic disease self-management, Patient engagement, Patient-centered care

## Abstract

**Background:**

Knowing how chronic kidney disease (CKD) patients talk about their encounters with providers (i.e., their discourse) can inform the important clinical goal of engaging patients in their chronic disease self-management. The aim of this study was to analyze patient discourse on ongoing CKD monitoring encounters for health communication strategies that motivate patient engagement.

**Methods:**

Passages regarding CKD monitoring from 6 focus group transcripts on self-management with a total of 30 participants age ≥ 70 years from the Atlanta Veterans Affairs Renal Clinic across three different CKD trajectories (stable, linear decline, and non-linear) were extracted. These passages were examined using three-stage critical discourse analysis (description, interpretation, explanation) for recurring patterns across groups.

**Results:**

Focus group participants were an average age of 75.1, 96.7% male, and 60% Black. Passages relating to CKD monitoring (*n* = 55) yielded predominantly negative communication themes. Perceived negative communication was characterized through a patient discourse of unequal exchange, whereby engaged patients would provide bodily fluids and time for appointments and continued to wait for meaningful, contextualized monitoring information from providers and/or disengaged providers who withheld that information. However, some encounters were depicted as helpful. Perceived positive communication was characterized by a patient discourse of kidney protection, whereby patients and providers collaborate in the mutual goal of preserving kidney function.

**Conclusions:**

Patient perceived an unequal exchange in CKD monitoring encounters. This perception appears rooted in a lack of easily understandable information. By accessing the positive discourse of protecting the kidneys (e.g., through eGFR level) vs. the discourse of damage (e.g., serum creatinine level), healthcare professionals can clarify the purpose of monitoring and in ways that motivate patient engagement in self-management. Patients being monitored for CKD progression may best be supported through messaging that conceptualizes monitoring as kidney protection and provides concrete contextualized information at each monitoring encounter.

## Background

Patients with chronic disease(s) are asked to adhere to self-management directives to limit disease progression, including regular monitoring visits for provider assessment, feedback and care-plan adjustments. These directives represent a shift from a traditionally passive to active disease management role for the patient and require new provider communication approaches that meet the needs of the patient in order to enable higher levels of patient engagement [1]. Knowing how patients talk about their encounters with providers (i.e., *their discourse*) can inform this goal.

The management of chronic kidney disease (CKD), in particular, presents special communication challenges. CKD is numerically communicated to patients in one of two ways, either in terms of damage to the kidney (albuminuria or the serum creatinine scale) or in terms of remaining kidney function (the estimated glomerular filtration rate (eGFR) scale) [2]. Clinical practice guidelines recommend patients follow a set of recommendations regarding medications and lifestyle, including regular monitoring visits to prevent further declines in eGFR, to identify CKD complications, and to prepare patients for transplant or dialysis if kidney failure occurs [2]. Among older adults, this situation is often complicated by an unpredictable (i.e., non-linear) trajectory of kidney function and the competing risk of death due to co-occurring chronic conditions before kidney failure occurs [3, 4]. Therefore, older adults may undergo routine CKD monitoring visits over many years without requiring treatment, progressing to kidney failure, or facing treatment decisions about dialysis or kidney transplant. Evidence suggests that CKD patients do not understand the goal of these regular monitoring visits, in part due to limitations in patients’ disease knowledge and in provider communications about CKD [5–7].

Suggested approaches to patient-centered communication include addressing the life context of the patient [8] or involving advanced practice providers [9] within the medical encounter, educating both providers and patients in ways to foster collaboration [10], and addressing health literacy [11]. A less-frequent approach is shaping communication messages from patient-provided discourse. Discourse analysis, or examining the ways that groups of people talk about topics, can identify practices that encourage or discourage behavior. A subset of discourse analysis, critical discourse analysis, has the potential to unearth deep normative parameters by which interactions are governed and open them up to intervention ([12], p. 28). We used critical discourse analysis to examine how patients talk about their CKD medical monitoring encounters in order to provide practical strategies for provider messaging in these encounters.

## Methods

The current study was based on a previous exploratory qualitative study designed to identify facilitators and barriers to older CKD patients’ self-management by CKD trajectory, details of which have been published elsewhere [3]. Potential focus group participants from the Atlanta Veterans Affairs (VA) Renal Clinic were mailed letters to announce the study and inform them that we would be contacting them by phone to determine their interest in participation in a focus group on self-management of CKD. Of 64 patients contacted by phone, 22 declined to participate due to lack of interest, transportation difficulty, or active health problems, two were ineligible due to low vision, eight had schedule conflicts and two scheduled but did not attend. Six focus groups that included 30 Veterans with CKD, age ≥ 70 years, were conducted in August and September 2014. All participants had moderate-to-severe CKD (at least one eGFR < 45 ml/min/1.73 m^2^) and were followed in Renal Clinic, a twice-weekly half-day clinic that focuses on the diagnosis, treatment, and monitoring of kidney diseases at the Atlanta VA Medical Center. This clinic is staffed by a team of nephrologists who have faculty appointments at Emory University, the local academic affiliate. Participants were not recruited from the separate VA Renal ‘Fellow Clinic.’ Groups were organized by kidney function trajectory determined from prior CKD monitoring visits: *stable*, *linear decline*, or *non-linear* [13, 14], ensuring the gamut of CKD experience within moderate-to-severe CKD. Prior to the focus group, participants individually completed Institutional Review Board approved informed consent and questionnaires that included validated brief instruments of health literacy, kidney disease self-efficacy, and perceptions of social support [14–18]. Focus groups followed a semi-structured interview guide, as is standard in focus group research [19], on the topic of self-management activities; the guide can be viewed in its entirety in the supplemental material for our published article [3]. Focus groups were recorded and recordings transcribed.

The current secondary analysis emerged from the observation of frequent, unexpected mentions of CKD monitoring encounters as particularly frustrating (an emergent theme). Driven by an interest in promoting patient-centered care in medically complex patients [20], we used critical discourse analysis, a socially engaged approach to discourse analysis, following Norman Fairclough [21]. Fairclough defined discourse as “a way of signifying a particular domain of social practice from a particular perspective.” In discourse analysis, the broad discourse that is the focus of analysis (e.g., patient discourse on CKD monitoring encounters) is analyzed for specific viewpoints or ways of thinking (named as particular “discourses”). Fairclough’s method entails examining text closely in three phases (see Table 1 for an example from our data). Verbatim transcripts from our six patient focus groups were read for references to CKD monitoring encounters (our target domain of social practice). These passages referred to or implied repeated encounters over time with any medical professional. Passages were extracted, analyzed by the lead author, and a spreadsheet of passages and codes shared with co-authors. Co-authors flagged passages where interpretation differed from their own. Differences were discussed weekly by the authorship team until differences were fully vetted, debated, and resolved. Following re-interpretation and adjustment at the three levels (description, interpretation, explanation), we looked for repeated patterns within and across the focus group, which we present below as “themes” (Table 1).

**Table 1:** Critical Discourse Analysis process demonstrated with exemplar CKD monitoring text passage

Textual passage ➜	Description ➜	Interpretation ➜	Explanation
	Noteworthy properties of the text, such as phonology, grammar, vocabulary, figures of speech, and organization, are enumerated to identify explicit and implicit meanings.	Features of discourse practice identified and interpreted in an interpersonal context.	Textual properties and discourse practice explained in relation to larger sociocultural practice, such as the VA or nephrology care.
I still haven’t been given any instructions, no treatment, or recommendations whatsoever. I am supposed to come back in 2 weeks and they are going to run some more lab tests again. You know basically I’ve been giving blood, five vials of blood, a urine sample every 3 months for the last, I don’t know, 5 or 6 years. And now they still said well no treatment. They said well we’ll know, I’ll see you in 2 weeks. I said now wait a minute, you gonna wait till I die to tell me? But anyway I’m sitting in a state now I have no medication. I haven’t been told to do anything in particular.	Statements about expected and actual patient actions in monitoring appointments are bracketed by statements about provider inaction in same	The Veteran speaker describes ongoing CKD monitoring visits to his nephrologist to fellow Veterans and the moderator/recorder.	Weighing actions of the patient against actions of the provider with the verb “give” conveys an expectation that the patient-provider encounter is an exchange governed by norms of reciprocity, with patient showing up to appointments and giving blood and urine and the providers giving meaningful information in return. Instead, here the exchange is presented as one-sided, with patient giving routinely and waiting for something but receiving nothing in return.
	Repeated use of word “still” and phrases “again” and “five or 6 years” emphasize duration of situation	Speaker’s juxtaposition of “I still haven’t been given” with “I’ve been giving” characterizes monitoring as an unequal exchange	
	Term “whatsoever” after statement about “instructions,” “treatment,” and “recommendations” from provider emphasizes totality of the lack	Addition of provider expectations (“I am supposed to”) suggests perceived double standard (expectations for patient but not for provider)	
	Phrase “I am supposed to” conveys perceived expectations placed on patient	Patient suggests that he is being strung along with the promise of information in the future	
	Verb “to give” used to describe both patient action (“I’ve been giving” blood and urine) and provider inaction (“I still haven’t been given”) brings patient and provider into direct comparison	Colloquial shift dramatizes confrontation in which patient rhetorically questions if information will come too late to help him	
	Provider talk summarized as “well, we’ll know, I’ll see you in 2 weeks” implies delayed but promised delivery of information		
	Phrase “Now wait a minute” indicates colloquial shift to directly addressing provider: “you gonna wait til I die to tell me?”		
	End phrase “sitting in a state now” suggests helpless passivity		
	Affective content is frustration		

## Results

**Fig. 1:**
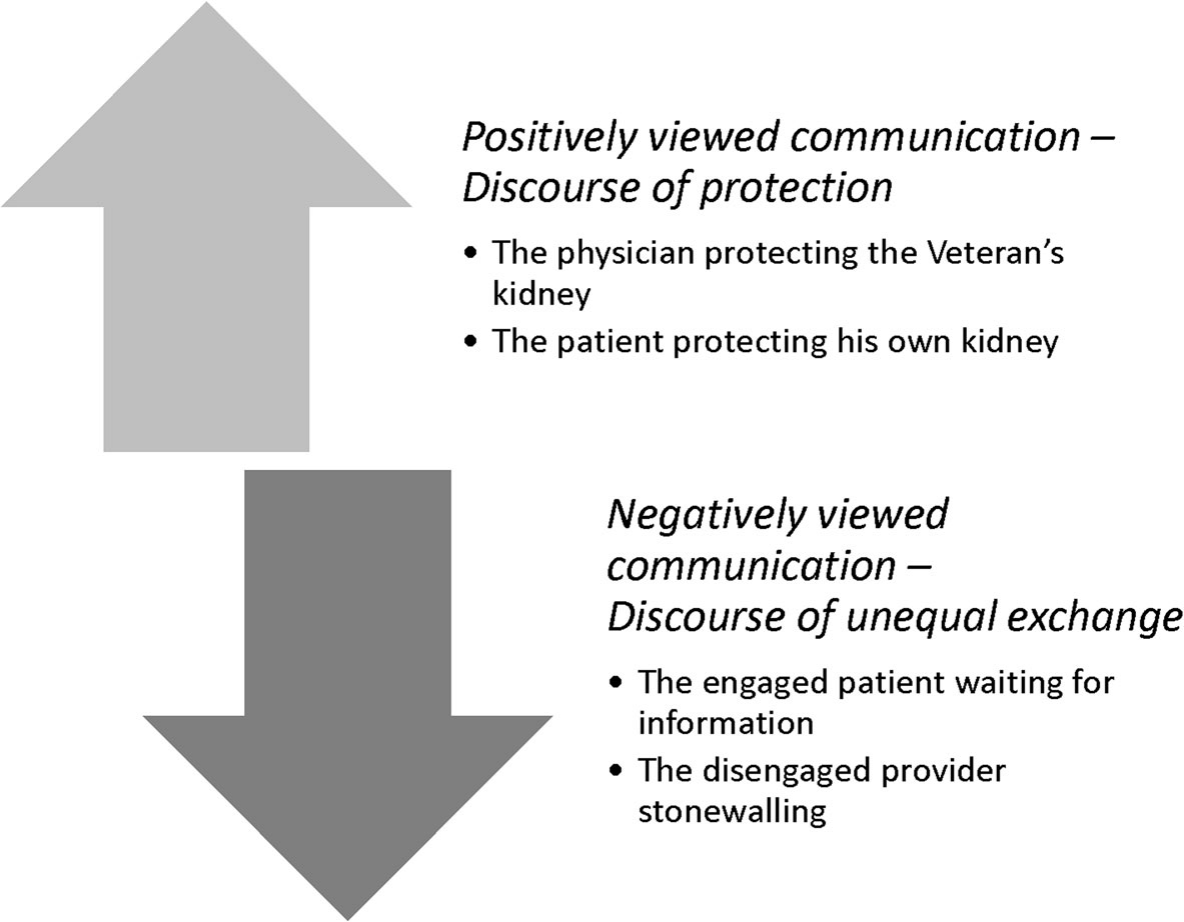
Patient-perceived positive and negative CKD monitoring communication

Focus group participants were an average age of 75.1, 96.7% male, and 60% Black. Inadequate health literacy ranged from 20% in the linear decline group to 27.3% in the non-linear trajectory group (Table 2). We identified 55 passages on physician-patient monitoring encounters. CKD patients expressed predominantly negative views, stressing that these encounters left them in a state of limbo, wary about their diagnosis and factors leading to CKD, confused about their role in CKD self-management, and uncertain about the future. In some cases, however, the encounters were depicted as helpful. The distinction between negatively and positively viewed encounters hinged upon the extent to which the goals of monitoring were understood. Where patients did not understand the purpose of monitoring, they tended to view encounters as unsatisfying and unsettling (Fig. 1).

**Table 2:** Characteristics of focus group participants by chronic kidney disease trajectory

Characteristics	Stable (n=9)	Linear decline (n=10)	Non-linear (n=11)
Age, mean (SD)	73.8 (3.1)	72.6 (6.5)	79.8 (4.1)
African-American race, n (%)	3 (33.3)	8 (80.0)	7 (63.6)
Male, n (%)	8 (88.9)	10 (100)	11 (100)
Income less than $20,000/year, n (%)	3 (33.3)	5 (50)	2 (18.2)
Inadequate health literacy^a^, n (%)	2 (22.2)	2 (20.0)	3 (27.3)
Confidence^b^, n (%)	11.0 (2.3)	10.5 (2.5)	10.0 (4.4)
Married, n (%)	6 (75.0)	5 (50.0)	4 (36.4)
Social support^c^, mean (SD)	20.0 (4.4)	19.5 (3.8)	19.0 (3.8)
Hypertension, n (%)	7 (77.8)	10 (100)	10 (90.9)
Diabetes, n (%)	2 (22.2)	10 (100)	8 (72.7)
Number of medications, mean (SD)	10.0 (5.1)	16.0 (6.0)	15.0 (6.9)
eGFR, ml/min/1.73 m^2^			
45 – 59	2(22.2)	0 (0.0)	2 (18.2)
30 – 44	6(66.7)	6 (60.0)	6 (54.6)
< 30	1(11.1)	4 (40.0)	3 (27.3)
Years of monitoring in renal clinic, median (range)	2.6 (0.7 – 8.3)	2.4 (0.3 – 7.5)	4.5 (0.9 – 12.9)

Trajectories included: *stable* (rate of decline < 2 ml/min/1.73 m^2^/year, total decrease not > 4.5 ml/min/1.73 m^2^, and no decline by > 8 ml/min/1.73 m^2^ between any 2 measurements), *linear decline* (consistent rate of decline > 4 ml/min/1.73 m^2^/year throughout available follow-up and total decline ≥ 8 ml/min/1.73 m^2^) and *non-linear* (all other trajectories). We adapted trajectory definitions from prior studies and available electronic health record eGFR data from the prior three years

^a^Health literacy screening defined as “somewhat, a little bit, or not at all” when asked “how confident are you filling out medical forms by yourself?” [14, 15, 17]

^b^Self-reported confidence in CKD related self-management tasks. Scores range from 6 to 30 with higher scores indicating lower confidence [16]

^c^Abbreviated social support measure. Scores range from 5 to 25 with higher scores indicating more support [17, 18]

### Negatively viewed communication – Discourse of unequal exchange

The negative and incomprehensible monitoring encounter was presented as an unequal exchange between the patient and the provider. This theme could be viewed from the separate roles of patient and provider.

#### The engaged patient waiting for information

The passage from a group with linear CKD decline in Table 1 lays out the basic features of CKD patient monitoring, in addition to presenting an unequal exchange. Monitoring for this patient requires regular periodic visits to a nephrologist, blood draws for creatinine testing, urine samples for albumin testing, and delayed communication about lab results.

A stable CKD trajectory patient described the experience of monitoring in terms of repeated visits at which he gets numeric results of the blood draws and urine labs, information that appears to be inadequate:

They of course checked the creatinine and found out that was 1.7 I think it was and went to see the – I don’t even know how the hell you pronounce it – went to see the kidney doctor and he changed some of the medicines I was on. Never told me anything about why I’ve got this high creatinine. To this day I don’t know, so I kept going back to see him every 6 months for 4 years. Finally he said, you don’t need to come back to see me anymore unless something changes, he said if you were to go to 2.5, I’d be concerned. Well I went up just over 2 a few months ago and that was the first time that I have ever been over 2 that I know of and it has come back down a little since then. I never had any ill effect whatsoever. I have no idea what it is.

The patient conveys distance from his health care providers through use of the pronoun “they” and his expletive in not being able to pronounce or say “nephrologist.” The superlative “never” having been given a causal explanation, as well as the unsettling disconnect between his diagnosis and the asymptomatic nature of the disease (“I never had any ill effect whatsoever. I have no idea what it is”) convey frustration. The phrase “to this day” indicates a long period of waiting, and he indicates that lack of information hooks him into repeat visits that lead nowhere. After 4 years, “finally” he receives a threshold where monitoring would shift concern, but he remains in limbo. The patient appears to lack any communication strategies for obtaining information on causal explanation, context, trajectory, and resolution. The serum creatinine scale cited is the only information that he has to work with in understanding his condition.

A non-linear CKD trajectory participant told fellow Veterans that sometimes the provider gave him:

…nothing. The last time I was in Renal Clinic, I went in, I sat down with my doctor, I had to go back to the time thing, well I’m like all service men, you’re taught to hurry up and wait, but it still gets on your nerves, I went in and waited for quite some time. ‘Ok, I’ll talk to your primary care doctor’; he didn’t tell me anything else.

Couched within a sense of service men having low status or being disrespected (“you’re taught to hurry up and wait”), a sense of irritation (“it still gets on your nerves”), and a sacrifice (“quite some time”), this Veteran’s apparent complaint is being cut out of the communication loop between his nephrologist and his primary care provider. According to the patient, the doctor provides little in exchange for his time and effort.

#### The disengaged provider stonewalling

Elaborating the theme of an unfair exchange, many Veterans presented providers as variously powerful, apathetic, or withholding of information during monitoring encounters. One non-linear CKD trajectory patient described seemingly benign, disengaged indifference to the monitoring process on the part of the physician:

When I go to the nephrologists, you know, they don’t really tell you very much the only thing that they usually do, is they test your, they take blood from you to find out what your creagnine (*sic*) is. Then they say “keep doing what you’re doing.” I’m at a 2.6, I think, it is a little high, but it is stable, “just keep doing what you’re doing,” And I only have 1 kidney. They, the nephrologists, don’t tell you that much. They have a nurse and they go in there and take your blood. Then they come in and say “things seem to be alright,” but things seem to be about the same. But I have a friend of mine and we were discussing his creagnine (*sic*), and it was maybe a point lower than me, and they put him on dialysis.

This Veteran describes a pattern of communication with several nephrologists. They or their nurses take and test blood and present serum creatinine levels. The repeated phrase “keep doing what you’re doing” does not satisfy the patient but leaves him wary, especially as a friend with an equivalent creatinine level initiated dialysis. A provider’s seemingly benign encouragement is experienced as an empty platitude. The serum creatinine number seems to confuse rather than clarify the situation for this Veteran.

A stable CKD trajectory participant indicated hearing the very same unhelpful phrase:

You know a lot of times if you see the doctor they say “Well, keep on doing what you doing, cause you seem to be doing better,” but you know… what is it that I am doing? Tell me something. But you know so you don’t know. Unless you get on the computer and do some research, or you have somebody around who can research things, to tell you well naw you don’t need to have that, you don’t need to have that, you just aren’t told anything.

This Veteran expresses how the phrase “keep on doing what you’re doing” is not only unhelpful (“what is it that I am doing?”) but also puts the burden entirely on patients, who may or may not have the skills to obtain information through their own research. He begs for his doctor to “tell me something.”

Other participants raised the issue of fragmented care as blocking the coherence and progress of their disease management. Two linear CKD decline patients agreed that CKD monitoring is part of care for multiple chronic conditions and that the patient is shuttled between various providers and treatments:

Participant A: She [primary care physician] started this kidney thing, she sent me because of my numbers moving to the renal doctor because of my numbers and she sent me to the cardiologist because of my heart failure. They put me on a certain medication, I’m on that medication for let’s say 4–5 months, because she doesn’t see me but every 6 months and she will say – she’ll sit there and look at the medication – ‘I see that they put you on so and so, I don’t like that,’ and she takes you off of it. Now how does that make you feel? You’re going to her specialist doctors. He’s taking you through all of the blood tests and puts you on a medication for 6 months, and then she says I don’t like that one and takes you off of it.

Participant B: Ping-pong. That’s what it is. It’s ping-pong.

Participant A: One of the things in your questionnaire was, do you get frustrated. That shoots your sugar up, shoots your blood pressure up.

Participant B sums up Patient A’s story as a game of “ping-pong” between doctors. We interpreted this statement in two ways: 1) that a game of table tennis is being played between providers with the patient as the ball, trying to keep up with iteratively changing treatment conditions, and 2) that the patient is a spectator of his own treatment, watching shifts in treatment direction, his presence almost incidental. Regardless of the interpretation, Participant A suggests that the general effect is frustration that worsens the patient’s underlying conditions of high blood pressure and diabetes. The core problem is lack of satisfying consideration and communication within the context of the patient’s overall health.

### Positive communication – Discourse of protection

Positive communication between patients and providers was depicted as comprehensible. The second discourse that emerged in the study was the discourse of protection, in which both patient and provider collaborate (with helpful give and take) in the mutual goal of protecting the kidney. As with the negative theme of unfair exchange, this theme could be viewed from the separate roles of patient and provider.

#### The veteran protecting his own kidney

Some Veterans conveyed commitment to monitoring and self-management because they understood the purpose of it. In helping their peers comprehend difficult aspects of CKD, such as lack of symptoms and the importance of regular monitoring, they framed the effort as preservation, an intuitive concept of the kidney that supported the idea of maintenance and monitoring. For example, a stable CKD trajectory Veteran explained to a peer that CKD monitoring was important to preserve what you’ve got uh with kidney function you do have, because it doesn’t get any better but at least you try to keep it from getting any worse, is the whole idea of it.... You got to have information on diet, the disease itself, general information and the medication. From what I understand there’s nothing that can make it better, you just want to keep it from getting worse.

The phrase “preserve what you’ve got” conveys the kidney as an important asset of the body, evoking other discourses of preservation such as self-preservation, historic preservation, and ecological preservation. The phrase “kidney function” connotes ongoing kidney filtration activity in positive terms.

A non-linear CKD trajectory patient organized all his self-management activities across several multiple chronic conditions around care for the heart:

The most dangerous thing is blood pressure for kidneys, that’s the most dangerous. If you keep your blood pressure under control that is better for your kidneys...That’s why you got to keep that blood pressure as level as you can.

Here the speaker identifies the kidneys as organs in danger, to be protected from that danger. Blood pressure “control” is a concrete and achievable goal.

A stable CKD trajectory patient presented CKD monitoring as comprehensible, using the implicit discourse of protection, in the following passage:

They said your creatinine has been pretty much stable and we’re just going to keep an eye on it…. They said 50%. I said how much does age contribute to it? And they said it might be something. I have been going to renal for about a year and a half. But ill effects, I really haven’t had any. 5, 6, 7 years, somewhere around there, and I had a physical and creatinine was elevated. So they started sending me to nephrology and I would go to nephrology first every 2 months, then finally every 6 months, the last time I went to VA nephrology, they said to come back in a year. They prohibit me from taking NSAIDs, anti-inflammatories and dietary restrictions. And again, I think it was started by high blood pressure.

The patient narrates the monitoring process without frustration. Care is orchestrated through a sequence of visits of decreasing frequency. He presents a back-and-forth dialogue about care during visits. There is clear causal explanation, instructions, and clear concept and goal of stabilization. One of the unique features of this passage is that it features the eGFR scale of monitoring. The patient notes that he still has “50%” function remaining.

#### The physician protecting the Veteran’s kidney

In the previous passage the patient indicates that doctors are going to “keep an eye on” his creatinine. The phrase suggests that the patient understands and accepts the role of his nephrologist in CKD monitoring as protecting the kidney over time. If the test results are significantly altered, it follows that the doctor and patient may need to adjust care. Although the patient uses the term “prohibit” in mentioning lifestyle restrictions, it is presented matter-of-factly, without the anger or frustration displayed within the discourse of unfair exchange.

A linear CKD decline patient steps in to help a frustrated peer by explaining the role of doctors: “What they mainly want to do is stabilize you. If you’re stabilized with the meds and all this stuff, you’re ok. As long as it doesn’t run away with you.” The word “stabilize” suggests work to eliminate volatility and continue in an uneventful, protected way, as in stabilizing patients in shock, buildings, war zones, and other dangerous situations. The purpose of monitoring is to control the situation and protect the kidney. Here the doctor is presented as a partner in working with the patient to keep the patient from getting worse. This patient also said, “If your numbers don’t get any higher, you could live to be 100 with kidney disease.” The concept of stabilization and protection is not only comprehensible but appealing.

Other patients praised the coordination provided by their primary care physician even as others regretted the lack of this coordination. Such coordination is presented as a clear contributory role in the Veteran’s care. As a non-linear CKD trajectory patient stated:

I trust my primary care physician that he has my best interest at heart, he knows what I’m taking, he’s getting the blood tests, he’s get the urine sample, he should know what I want, if I got a problem...he’s willing to send me to a specialist and find out if I need anything or not.

Within the discourse of protection, patient and provider are aligned around the same goal, stabilize and protect the kidney, which appears comprehensible to these patients.

## Discussion

Analysis of discourse by Veterans ≥70 years old who were actively monitored for progression of moderate-to-severe CKD indicated that when patients did not understand the purpose of monitoring, they tended to view encounters as unsatisfying and unsettling. These patients complained that they did not hear from their providers why they were there, they did not understand the purpose of monitoring, and they did not know how to “keep doing what you’re doing.” Participants who did not understand the purpose of monitoring accessed a negative discourse of unequal exchange. Such participants weighed perceived actions of patients against perceived actions of nephrologists and felt that the latter came up short. In this imbalanced equation, patients were repeatedly showing up for appointments, providing blood and urine, and waiting for meaningful information on their condition. Nephrologists, according to this view, were disengaged, unaware of their patient’s information need, unable to provide it, or unwilling to do so. As one of our dissatisfied Veteran participants grumbled, “I give them my blood, and they don’t tell me anything.”

Theoretically, Veteran CKD patients’ frustration at receiving inadequate information can be explained by the implied cultural norm of reciprocity [22], in which both parties in a social exchange give and receive equally over time. Older Veterans who depict themselves as making an effort to come to monitoring appointments, give time, blood, and urine, sometimes for years, while still waiting for quality information in return, suggest an accruing debt to the patient on the part of the provider. One risk of this perceived debt, if unpaid for too long, is disengaged and cynical patients. Veterans’ sense of unequal exchange and perceptions of unfairness may be couched within a broader distrust of the VA system [23]. We acknowledge that the VA had received negative publicity about patient wait times prior to the focus groups that may also have made our participants’ frustrations more salient. Nevertheless, systematic reviews suggest that VA performs similarly or better than non-VA settings specific to processes of care quality [24, 25]. Lederer and colleagues [26] conducted a qualitative study of barriers contributing to Veterans’ unmet CKD information needs, reporting that barriers to patient-provider communication were evident based on the VA and non-VA care experiences of Veterans with CKD. Additional research in community settings to further elucidate the experience of CKD monitoring and associated communication in more varied, comparative studies is needed. Such work could determine, for instance, whether patient frustration with CKD monitoring communication is widespread and identify individual and system level determinants of such experience.

In practice, findings from the current study suggest the importance of conceptualizing test results in terms of remaining function, continuous reinforcement through repetition, and providing a tangible information takeaway at each monitoring encounter. How kidney functioning is conceptualized in language influences CKD patient perceptions of kidney monitoring encounters. It is possible that providers are not so much disengaged as lacking the skills to communicate effectively with patients. Informational needs in CKD patients are known to include general knowledge about kidney disease, natural history, sensitivity and specificity of screening/diagnostic tests, and condition management [27]. The National Kidney Foundation’s scale for diagnosing, tracking, and managing CKD (using an estimating glomerular filtration rate (eGFR) and presenting the amount of kidney function remaining from the CKD threshold of < 60 to the kidney failure threshold of < 15) is preservation-oriented and seemed to fit with our Veteran discourse of protection. In our older patient sample, the eGFR scale calibrated in terms of remaining kidney function appears easier for patients to grasp than the inverse serum creatinine scale calibrated on existing damage and failure. One underlying reason could be the positivity effect in aging, whereby older adults preferentially attend to and remember positive over negative information in contrast with young adults due to a shorter future time horizon [28]. Although eGFR reporting is now routine at the VA [29, 30], the frequent mention of the serum creatinine number in our sample suggests that providers more commonly frame lab results in terms of damage.

Further, our participants suggested that patients may need continuous reinforcement of the purpose of monitoring blood- and urine-based lab results. This need might be met through consistent contextualized information about CKD monitoring results during each monitoring encounter. As an example, the National Kidney Disease Education Program offers a patient-education pad called *How Well Are Your Kidneys Working?* for use in clinical interactions with tear-off sheets and easy-to-understand language about eGFR, with a blank space to insert the most recent lab result [31], a vetted tool that was rated highly in terms of reading level (6th grade), message content, visuals, and layout [32, 33]. The handout visualizes the kidney as a gas tank that is less than full. The gas tank image conveys the goal of keeping the tank as full or functional as possible. Providing a tangible information sheet with each encounter may display both effort to reciprocate to patients by giving them information in medical situations where medicine or advice is not always necessary while also reinforcing the meaningful context of protecting kidney function over time. Useful patient-centered tools exist (e.g., [26]) but we do not know how widely specific tools are implemented in practice.

Our secondary analysis of a study focused on CKD self-management was not based on any specific questions about CKD monitoring. Rather our data emerged from spontaneous, unsolicited focus group comments and discussion. These data were sufficiently rich and compelling to urge the examination of the additional research question suggested by the CKD self-management data and then pursue analysis, with meaningful results.

The results of this secondary qualitative study have several inherent limitations. Since the study was not specifically conceptualized to investigate the experience of CKD monitoring communication, we were unable to ascertain thematic saturation. Different themes may have emerged had participants been asked directly about communication in CKD monitoring encounters. Future, dedicated investigations of CKD monitoring communication would likely generate additional themes, beyond those identified here, to explicate further the CKD monitoring experience of CKD patients. For instance, individual differences may drive variations in the experience of CKD monitoring and associated communication. Our focus group data did not encompass contextual information about CKD monitoring visits from which we could discern social relations between actors in the broader sociocultural context. Our data were analyzed by a single investigator, the lead author, and discourse analysis is inherently subjective despite checks and balances through group discussion. Our study was limited to one VA site, serving a select population that is predominantly male. Steps were taken to recruit participants with a broad range of experiences to encourage discussions about CKD self-management. We did not, however, assess satisfaction with renal care or probe to determine if focus group contributions were a reflection of underlying frustration with VA care. We cannot know if patients who were frustrated with VA care may have been more likely or less likely to volunteer their time as research participants, However, our participants were older Veterans who were most likely to be struggling with self-management of multiple chronic conditions. These data represent the experiences and views of patients with CKD, but much remains to be understood about the larger context of CKD monitoring communication, including providers’ experiences and viewpoints.

## Conclusions

CKD patients’ need for both comprehensible information and for reciprocity in the monitoring encounter may best be supported by maximizing positive communication through provider messages that emphasize kidney protection and by minimizing perceptions of unequal exchange by providing consistent and contextualized information about CKD monitoring results during each monitoring encounter.

## Abbreviations

CKD: Chronic kidney disease
eGFR: Estimated glomerular filtration rate
